# Accuracy of conventional urinary output monitoring in the ICU

**DOI:** 10.1186/cc10837

**Published:** 2012-03-20

**Authors:** E Bouwhuijsen, A Oude Lansink, MW Nijsten, W Dieperink

**Affiliations:** 1 University of Groningen, University Medical Center Groningen, the Netherlands

## Introduction

In patients who are treated in the ICU an accurate fluid balance is an important tool to assess their hydration status. In most ICUs, intake of fluid is monitored precisely by sophisticated volumetric infusion and feeding pumps. In contrast to fluid intake, fluid output - especially urine as its most important component - is usually monitored visually by hourly assessment of the amount of fluid lost and urine production. Thus measurement of urinary output is a repetitive procedure 24 times a day which requires handling of the urinary collection system, visual assessment and manual data recording, actions that are easily affected by human errors.

## Methods

In a bench test we investigated the accuracy and precision of conventional urinary output monitoring, by visual hourly readings and manual data recording, as performed by experienced intensive care nurses with the purpose to provide insight into potential errors in urinary output measurement as well as identifying systematic sources of error. Two different types of ordinary 24-hour urine meters were used. The meters were filled with a predetermined amount (gold standard) of yellow lemonade. Both urine meters were filled with variable but identical volumes for a range of 8 to 325 ml, to a total amount of 3,600 ml. Hereafter the nursing staff manually recorded the reading of 48 prefilled urine meters.

## Results

Forty-eight nurses performed 2,285 urine volume measurements in two different types of ordinary urine meters (Bard Urine meter drainage bag; Bard Medical, Covington, Georgia, USA and Rüsh U-bag; Jiangsu, People's Republic of China). The mean measured output for the Bard urine meter was 3,688 ml, SD ±45 and for the Rüsh urine meter 3,692 ml, SD ±55. The limits of agreement between both types of urine meters were 2.4% to 2.6% respectively. Compared with the gold standard, analysis demonstrated deviations of 2.6% for both types of urine meters.

## Conclusion

Conventional urinary output measurement with ordinary urine meters constitutes a simple and accurate method for measuring urine volume in the ICU.

